# Elevated serum C1q is an independent predictor of high residual platelet reactivity in CAD patients receiving clopidogrel therapy

**DOI:** 10.3389/fimmu.2022.969984

**Published:** 2022-09-30

**Authors:** Zehao Zhao, Meishi Ma, Xin Huang, Tienan Sun, Kangning Han, Shiwei Yang, Yujie Zhou

**Affiliations:** ^1^ Department of Cardiology, Beijing Anzhen Hospital, Capital Medical University, Beijing, China; ^2^ Beijing Institute of Heart, Lung and Blood Vessel Disease, Beijing, China; ^3^ Beijing Key Laboratory of Precision Medicine of Coronary Atherosclerotic Disease, Clinical center for coronary heart disease, Capital Medical University, Beijing, China

**Keywords:** clopidogrel, complement C1q, platelet activity, thromboelastography, percutaneous coronary intervention, coronary artery disease

## Abstract

**Background:**

Inflammation increases the risk of thrombosis in coronary artery disease (CAD) patients and affects the antiplatelet efficacy of clopidogrel. C1q interacts with platelets to activate platelets and induce thrombosis by participating in and regulating the inflammatory response. Whether C1q affects adenosine diphosphate (ADP)-induced platelet reactivity during clopidogrel therapy was unclear and our study aimed to explore the issue.

**Method:**

We enrolled 1,334 CAD patients receiving clopidogrel therapy and evaluated the association between C1q level and high residual platelet reactivity (HRPR) using logistic regression and restricted cubic spline (RCS). HRPR was defined as ADP-induced maximum amplitude (MA_ADP_) > 47 mm plus ADP-induced platelet aggregation (ADP_i_) < 50%.

**Results:**

A total of 516 patients (38.7%) performed HRPR. The frequency of HRPR increases with the increase in C1q level (26.3%, 38.4%, 43.2%, and 46.7% for the 1st to 4th quartile of C1q). The result of multivariate logistic regression demonstrated elevated C1q as an independent predictor for HRPR (2^nd^ quartile: OR = 1.722, 95% CI 1.215–2.440; 3^rd^ quartile: OR = 2.015, 95% CI 1.413–2.874; 4^th^ quartile: OR = 2.362, 95% CI 1.631–3.421, compared to the 1st quartile). RCS depicted the nonlinear relationship between C1q and HRPR risk (*p* for non-linear < 0.05).

**Conclusion:**

The current research is the first to explore the association of C1q and ADP-induced platelet reactivity and to demonstrate elevated C1q as an independent risk factor for HRPR in CAD patients during clopidogrel therapy.

## Introduction

As the leading cause of death worldwide, cardiovascular diseases represent more than 40% of all deaths among Chinese (1). CAD, usually caused by atherosclerosis, is one of the most severe cardiovascular diseases, which often results in myocardial dysfunction and/or organic lesions. Percutaneous coronary intervention (PCI) is the major revascularization strategy for CAD patients with clinical indications for improving current myocardial blood supply and long-term outcomes. Dual antiplatelet therapy (DAPT) consisting of aspirin and oral P2Y12 inhibitors represents a guideline-recommended cornerstone of secondary prevention in patients undergoing PCI (2).

Clopidogrel, one of the earlier P2Y12 inhibitor agents to be applied (3), is still widely applied currently in peri-interventional treatment and long-term DAPT after PCI among patients with stable coronary artery disease (SCAD) (2, 4), especially on those at relatively lower risk. Clopidogrel possibly decreases bleeding risk compared with ticagrelor and prasugrel (5, 6) in acute coronary syndrome, especially in elderly patients (7). The antiplatelet efficacy of clopidogrel is influenced by multiple factors like drug interactions, genetic polymorphisms, and clinical and biological factors (8). As a result, a clinically significant proportion of patients treated with recommended doses of clopidogrel cannot the display desired antiplatelet response (9). Previous studies had demonstrated that HRPR during clopidogrel therapy is associated with adverse ischemic events, particularly in early stent thrombosis (10).

Complement component C1q is the recognition molecule of the classical activation pathway, which plays a critical role in defending against invading pathogens, maintenance of immunologic tolerance, and modulation of inflammatory responses (11). Serum level of C1q had been proposed as a biomarker for diagnosis and assessing activity in certain diseases (12). The classical complement pathway is involved in the regulation of atherosclerosis progression and exerts dual effects in different stages (13), and both low and high C1q plasma levels can be potential risk factors for CAD (14–16). Previous research had indicated that inflammation induces plaque rupture and coronary thrombus formation (17), and influences ADP-induced platelet aggregation during clopidogrel treatment. C1q and classical pathway also interact with platelets and coagulation factors to promote hemostasis and thrombotic processes by participating in and regulating inflammatory response (16, 18), and activation of the classical pathway was observed in acute coronary thrombi (19). However, whether C1q affects the antiplatelet efficacy of clopidogrel remains unknown. The present investigation collected clinical data from SCAD patients who had undergone elective PCI to compare the differences in clinical characteristics between individuals with and without ADP-induced HRPR and aimed to explore the contribution of C1q on ADP-induced HRPR during clopidogrel therapy.

## Methods

### Study design and patients

**Figure 1:**
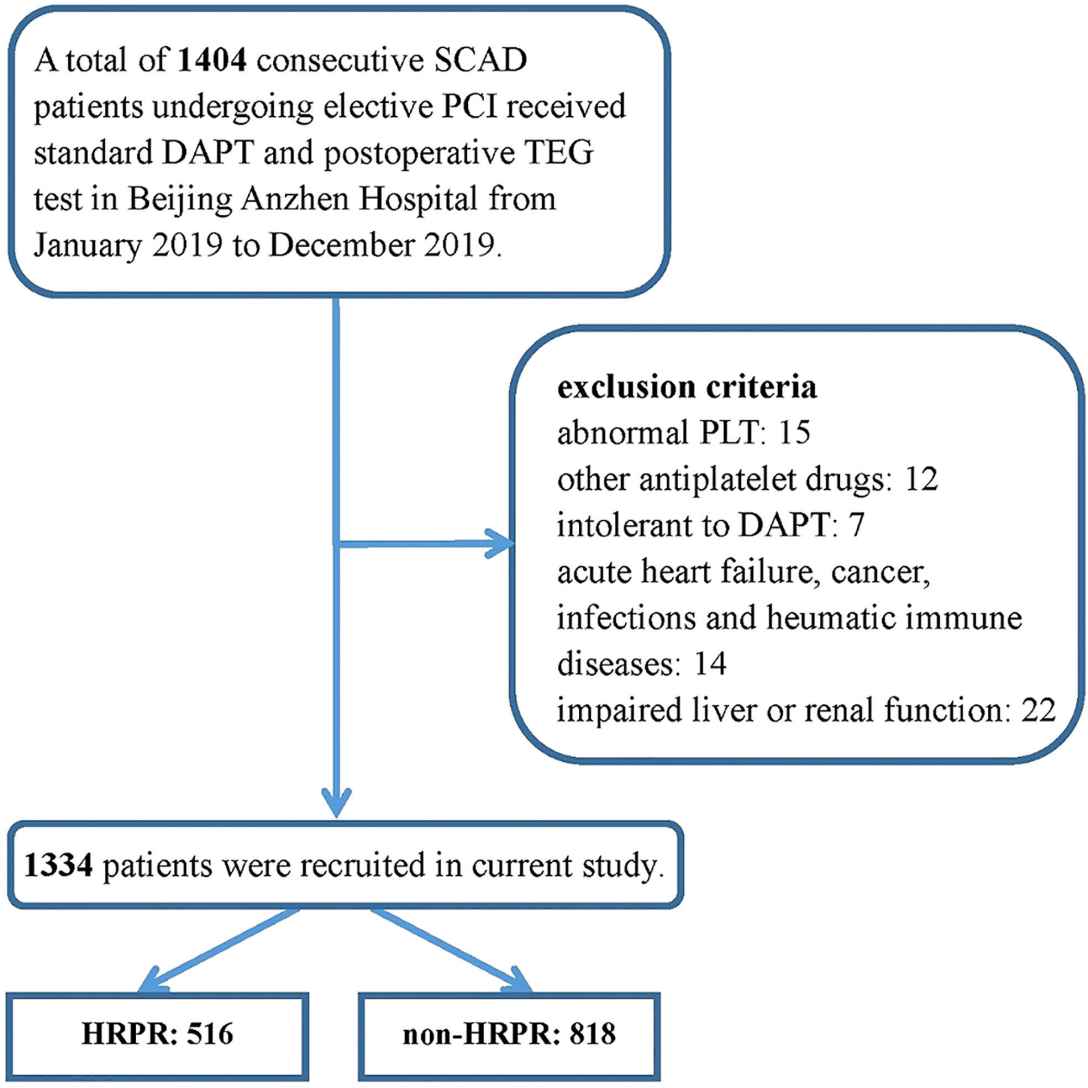
Inclusion and exclusion in the study population.SCAD, stable coronary artery disease; PCI, percutaneous coronary intervention; DAPT, dual antiplatelet therapy; TEG, thromboelastography; PLT, platelet count.

A cross-sectional study was carried out to explore the association between C1q and ADP-induced HRPR during clopidogrel therapy. Study data were derived from a prospectively collected database, which was created in the Twelfth Department of Cardiology, Beijing Anzhen Hospital, Capital Medical University. We consecutively recruited 1,404 SCAD patients undergoing elective PCI prospectively and evaluated their platelet function using thromboelastography (TEG) from January 2019 to December 2019. All the subjects were Chinese Han population of northern China. All participants were required to receive a loading dose (300 mg) of clopidogrel at least 12 h before PCI or receive a maintenance dose (75 mg, once daily) for at least 5 days before PCI. The following were the exclusion criteria (1) age <18 years old, (2) an abnormal baseline platelet count of <50 × 109/L, or >400 × 109/L, (3) taking other drugs known to affect platelet function, (4) intolerant to DAPT therapy consisting of aspirin and clopidogrel [e.g. BARC type 3 to 5 bleeding (20) or drug allergy], (5) presence of acute heart failure, (6) presence of acute or chronic infections, (7) known history of rheumatic immune diseases, (8) with neoplastic diseases, and (9) severely damaged renal or/and liver function (estimated glomerular filtration rate < 30 ml/min/1.73 m2, alanine aminotransferase > 2.5 times the normal upper limit). The details of inclusion and exclusion criteria are shown in **Figure 1**.

### Demographic, clinical, and laboratory information

We collected and documented every patient’s information according to the unified standard process. Data entry clerks collected demographic, clinical, and laboratory information from Beijing Anzhen Hospital’s electronic medical record management system and constructed a database. Demographic variables consisted of gender and age. Clinical variables included heart rate, blood pressure, body mass index (BMI), smoking history, and previous medical history. BMI was calculated as weight (kg)/[height(m)]^2^. Patients with BMI >30 kg/m^2^ were classified as obese population. Patients’ smoking status was self-reported, which was grouped into current (smoking within the last 3 months), former (quitted for more than 3 months before admission), and never smokers. Hypertension was defined as the previous history, use of antihypertensive medication, or current diagnosis (≥140/90 mmHg more than two times on different days). Diabetes was diagnosed according to the previous history of diabetes, current diagnosis based on the guidelines (21), or those who received a prescription of either an oral hypoglycemic agent or insulin. Patients with dyslipidemia were defined as those with total cholesterol (TC) > 6.2 mmol/L, high-density lipoprotein cholesterol (HDL-C) < 1.0 mmol/L, low-density lipoprotein cholesterol (LDL-C) > 4.1 mmol/L, or triglyceride (TG) > 2.3 mmol/L. Cerebrovascular disease included cerebral bleeding or ischemic attack. The history of revascularization was established based on patients’ medical records.

All of the patients included in our study were examined in the same way. The peripheral venous blood sample was drawn after at least 8 h of fasting. Laboratory data included routine blood indicators [hemoglobin (Hb), platelet count (PLT), and white blood cell count (WBC)], lipid profiles (HDL-C, LDL-C, TC, and TG), glycemic parameters [glycosylated hemoglobin A1c (HbA1c) and fasting blood glucose (FBG)], uric acid (UA), creatinine (CR), albumin (ALB), aspartate aminotransferase (AST), alanine aminotransferase (ALT), high-sensitivity C-reactive protein (hs-CRP), and complement C1q, which were obtained from the Department of Laboratory Medicine of Beijing Anzhen Hospital based on standard procedure. We used an online tool (http://ckdepi.org/equations/gfr-calculator/) to get an estimated glomerular filtration rate (eGFR) based on Chronic Kidney Disease Epidemiology Collaboration (CKD-EPI) equation. We used a biochemical analyzer to test serum C1q based on the operation process and quality control criteria suggested by the instrument’s instruction (Hitachi-7600, Tokyo, Japan).

### Assessment of platelet aggregation

We used the LEPU TEG System (CFMS, Beijing, China) to assess ADP-induced platelet aggregation, the detection principle of which was described in detail in previous literature (22, 23). Samples of fasting blood were taken from the patients on the following morning after PCI and processed within 2 h. The equipment adds 1 ml of heparinized blood into a vial containing kaolin, and then transfers 500μl of activated blood to a vial containing heparinase in order to neutralize heparin. Next, the equipment immediately adds 360 μl of neutralized blood into a cup coated with heparinase and measure the maximum amplitude of thrombin-induced clot strength (MA_thrombin_) by the TEG System. Then, the equipment adds 340 μl of heparinized blood into a cup with activator F and reptilase to measure the maximum amplitude of fibrin cross-linked clot without platelet (MA_fibrin_). Finally, the equipment adds 340 μl of heparinized blood to a cup coated with nonheparinase in the presence of the activator F and ADP (2 μmol) to generate a whole blood cross-linked clot with activated platelet and measure its max strength (MA_ADP_). MA_ADP_ reflects the peak intensity of clot developed by ADP in a heparinized whole blood sample, which represents the aggregation capacity of platelet and fibrin induced by ADP. The aggregation capacity of platelet and fibrin induced by thrombin and the aggregation capacity of only fibrin induced by activator F is evaluated by the maximum amplitude of thrombin-induced clot strength (MA_thrombin_) and of fibrin clot strength (MA_fbrin_), respectively. The efficacy of clopidogrel in inhibiting ADP-induced platelet aggregation was assessed using ADPi, which was calculated as ${\text{ADP}}_{\text{i}}=\frac{\left({\text{MA}}_{\text{ADP}}–{\text{MA}}_{\text{Fibrin}}\right)}{\left({\text{MA}}_{\text{Thrombin}}–{\text{MA}}_{\text{Fibrin}}\right)}\times 100\%$ . ADP-induced HRPR during clopidogrel treatment was defined as MAADP > 47 mm plus ADP-induced platelet inhibition rate < 50% (24, 25).

### Statistical methods

We presented continuously normally or non-normally distributed variables by the mean and standard deviation (SD) or by the median and interquartile range (IQR), and presented categorical variables by number and percentage. Normality distribution was assessed using graphical methods and the Shapiro–Wilk test. To compare differences between groups with low and high responses to clopidogrel, p-values were determined using the Student’s t-test or Mann–Whitney test for continuous normally or non-normally distributed variables and using the chi-square test for categorical variables. C1q level was divided into quartiles, and we detected baseline characteristics’ differences among patients with different quartiles of C1q using analysis of variance, Kruskal–Wallis test, or chi-square test when appropriate. Chi-square test, Student’s t-test, and Mann–Whitney test were used to multiply assess differences in ADP-induced platelet aggregation between the reference group (the lowest quartile of C1q) and each higher level using a Bonferroni-corrected significance level of p = 0.05/3. We calculated the odds ratio (OR) and 95% confidence interval (CI) for HRPR in each C1q quartile using univariate logistic regression analysis. In order to evaluate the effect of C1q on a continuous scale, we additionally constructed models including continuous C1q. Furthermore, we included a series of confounders that were picked based on clinical judgment, previous literature, and statistical significance, in multivariable regression models while correcting for confounders (1) Model 1: adjusted for age and sex (female); (2) Model 2: adjusted for variables included in Model 1 and smoking status (current smoker), and medical history of diabetes and hypertension; (3) Model 3: adjusted for variables included in Model 2 and laboratory data including PLT, WBC, Hb, LDL-C, HDL-C, eGFR, and hs-CRP]. To reduce the risk of distorted model, we additionally calculated the variance inflation factor (VIF) for each covariate to test data multicollinearity. All models met our criteria of nonmulticollinearity with VIF less than 5 (**Supplementary Table 1**). A linear trend test across quartiles was performed by assigning medians to each quartile of C1q as a continuous variable in these models. We also used restricted cubic splines (RCSs) fitted for univariate and multivariable regression models with three knots at the 25th, 50th, and 75th percentiles to flexibly model and visualize the nonlinear relation between C1q and HRPR risk. We set the median of C1q’s first quartile as the reference. A likelihood ratio test comparing the RCS model with a model including only a linear term was used to test for potential non-linearity.

A two-tailed p-value of < 0.05 was considered significant. All statistical analyses were performed using SPSS Statistics 26.0 (SPSS, Inc., Chicago, IL, USA) and R (v. 4.0.5).

## Results

### Baseline characteristics

After screening according to inclusion and exclusion criteria, a total of 1,334 participants were consecutively recruited in this study, of whom 516 patients (38.7%) had low response to clopidogrel. All covariate data of interest are complete. Baseline characteristics are presented using stratification based on low and high ADP-induced platelet aggregation in **Table 1**. Patients with HRPR are older and have a higher proportion of female patients, compared to the controls. The proportion of current or former smokers in patients with HRPR is lower, which reaches borderline statistical significance (p = 0.053). Patients with HRPR are more likely to have a medical history of hypertension (p = 0.053) and diabetes and have lower levels of WBC, Hb, and eGFR as well as higher levels of PLT, LDL-C, HDL-C, hs-CRP, and C1q (178.49 ± 31.11 mg/L vs. 168.56 ± 31.57 mg/L, p < 0.001) at baseline laboratory data.

**Table 1:** Baseline characteristics in patients with and without HRPR.

Variables	Overall	Patients without HRPR	Patients with HRPR	*p*
*N* (%)	1,334	818 (61.3%)	516 (38.7%)	-
**Clinical data**				
Female, *n* (%)	361 (27.1%)	158 (19.3%)	203 (39.3%)	<0.001
Age (years), mean ± SD	61.28 ± 9.53	60.35 ± 9.656	62.75 ± 9.130	<0.001
BMI (kg/m^2^), mean ± SD	25.93 ± 6.05	25.82 ± 3.20	26.10 ± 8.86	0.417
Obesity, *n* (%)	125 (9.4%)	71 (8.7%)	54 (10.5%)	0.276
HR (bpm), mean ± SD	69.55 ± 7.64	69.61 ± 7.61	69.45 ± 7.69	0.703
SBP (mmHg), mean ± SD	129.04 ± 15.86	128.43 ± 15.91	130.00 ± 15.75	0.079
DBP (mmHg), mean ± SD	75.35 ± 10.99	75.55 ± 10.92	75.03 ± 11.10	0.399
Smoking				0.053
Smoker, *n* (%)	343 (25.7%)	228 (27.9%)	115 (22.3%)	
Ex-smoker, *n* (%)	92 (6.9%)	51 (6.2%)	41 (7.9%)	
Never-smokers, *n* (%)	899 (67.4%)	539 (65.9%)	360 (69.8%)	
**Medical history**				
Hypertension, *n* (%)	828 (62.1%)	491 (60.0%)	337 (65.3%)	0.053
Diabetes, *n* (%)	470 (35.2%)	267 (32.6%)	203 (39.3%)	0.013
Dyslipidemia, *n* (%)	638 (47.8%)	394 (48.2%)	244 (47.3%)	0.754
Previous cerebrovascular disease, *n* (%)	115 (8.6%)	65 (7.9%)	50 (9.7%)	0.269
Previous PCI, *n* (%)	384 (28.8%)	250 (30.6%)	134 (26.0%)	0.071
Previous CABG, *n* (%)	31 (2.3%)	17 (2.1%)	14 (2.7%)	0.453
**Biochemical data**				
WBC (×10^9^/L), mean ± SD	6.72 ± 1.89	6.88 ± 1.95	6.45 ± 1.75	<0.001
Hb (g/L), mean ± SD	140.52 ± 15.97	143.11 ± 15.68	136.42 ± 15.57	<0.001
PLT (×10^9^/L), mean ± SD	212.71 ± 3.86	205.88 ± 53.82	223.55 ± 52.18	<0.001
ALT (mmol/L), median [IQR]	20.00 [15.00–29.00]	21.00 [15.00–29.00]	20.00 [15.00–30.00]	0.194
AST (mmol/L), median [IQR]	21.00 [18.00–25.00]	21.00 [18.00–25.00]	21.00 [17.25–26.00]	0.697
ALB (g/L), mean ± SD	42.31 ± 3.59	42.31 ± 3.45	42.32 ± 3.79	0.973
TC (mmol/L), mean ± SD	4.01 ± 0.98	3.94 ± 0.96	4.11 ± 1.01	0.003
LDL-C (mmol/L), mean ± SD	2.34 ± 0.80	2.30 ± 0.79	2.40 ± 0.82	0.027
HDL-C (mmol/L), mean ± SD	1.09 ± 0.25	1.07 ± 0.24	1.11 ± 0.26	0.027
TG (mmol/L), mean ± SD	1.62 ± 1.19	1.65 ± 1.29	1.59 ± 1.01	0.411
FBG (mmol/L), median [IQR]	5.65 [5.06–6.96]	5.63 [5.01–7.00]	5.72 [5.12–6.91]	0.097
HbA_1_C (%), median [IQR]	6.20 [5.80–7.00]	6.20 [5.70–6.90]	6.30 [5.80–7.10]	0.059
UA (μmol/L), mean ± SD	348.66 ± 86.62	350.05 ± 82.79	346.47 ± 92.41	0.474
Creatinine (μmol/L), mean ± SD	71.70 ± 18.24	72.29 ± 17.15	70.77 ± 19.82	0.152
eGFR (ml/min/1.73 m^2^), median [IQR]	94.57[85.90–101.23]	95.09 [86.93–102.21]	93.41 [84.46–99.96]	0.001
Hs-CRP (mg/L), median [IQR]	1.18 [0.50–2.93]	1.05 [0.44–2.61]	1.4300 [0.69–3.47]	<0.001
C1q (mg/L), mean ± SD	172.41 ± 31.75	168.56 ± 31.57	178.49 ± 31.11	<0.001
**TEG parameter**				
MA_ADP_ (mm), median [IQR]	43.45 [34.98–51.30]	37.10 [29.40–41.70]	53.70 [49.80–58.40]	<0.001
ADPi (%), mean (SD)	37.15 ± 23.60	50.49 ± 19.38	15.99 ± 10.66	<0.001

HRPR, high residual platelet reactivity; PCI, percutaneous coronary intervention; CABG, coronary artery bypass graft; BMI, body mass index; HR, heart rate; SBP, systolic blood pressure; DBP, diastolic blood pressure; PLT, platelet count; Hb, hemoglobin; WBC, white blood cell count; ALT, alanine aminotransferase; AST, aspartate aminotransferase; ALB, albumin; TC, total cholesterol; TG, triglyceride; LDL-C, low-density lipoprotein cholesterol; HDL-C, high-density lipoprotein cholesterol; FBG, fasting blood glucose; HbA_1_C, hemoglobin A_1_C; UA, uric acid; CR, creatinine; eGFR, estimated glomerular filtration rate; hs-CRP, high-sensitivity C-reactive protein; MA_ADP_, maximum amplitude of ADP-induced clot strength; ADP_i_, ADP-induced platelet inhibition rate.

### Correlations between C1q and other variables

**Figure 2:**
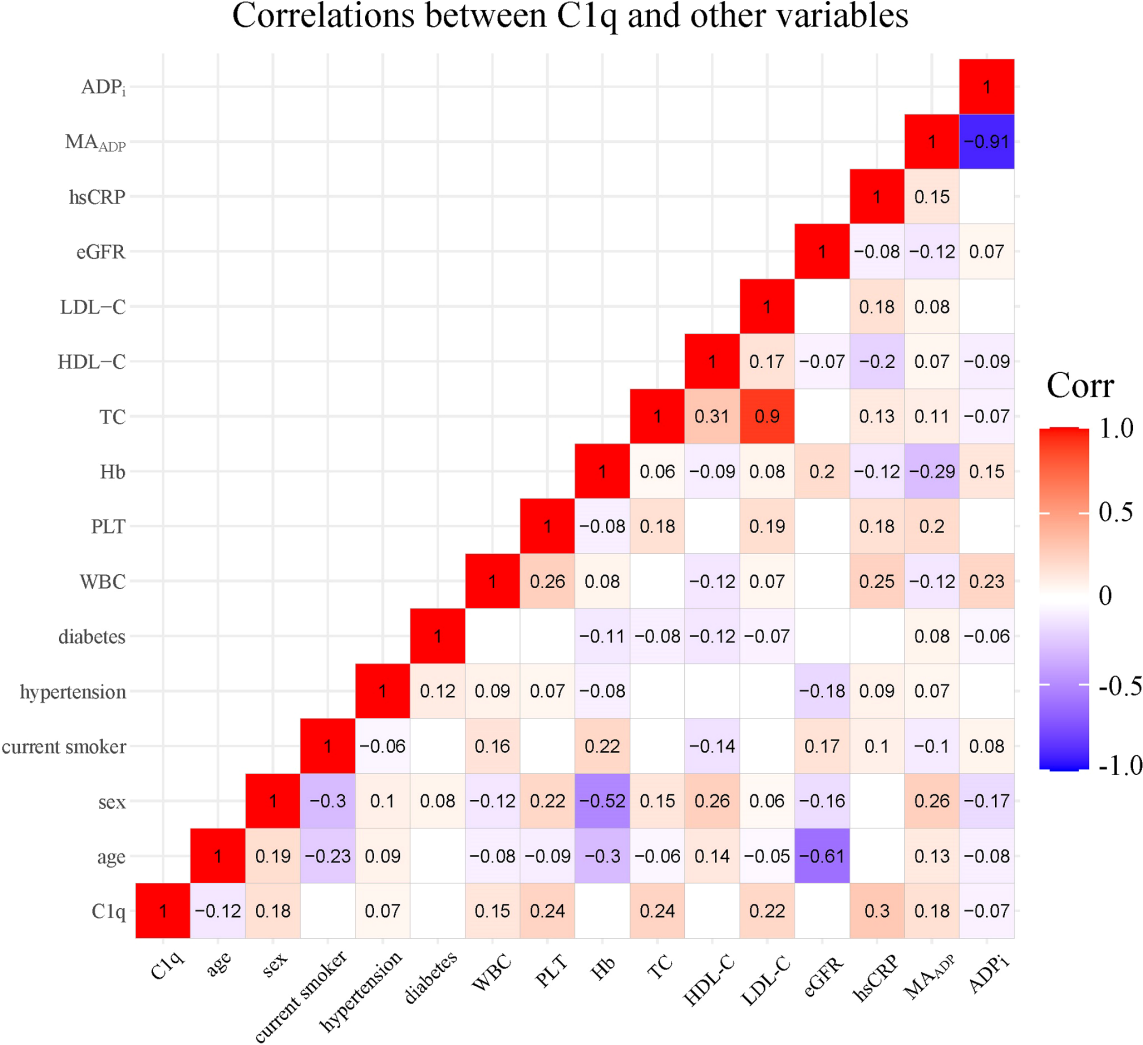
Correlations between C1q and covariates of interest. The number and color in each box represented correlation coefficient for two covariates corresponding to the box, of which blank indicated non-statistically significant association. PLT, platelet count; Hb, hemoglobin; WBC, white blood cell count; TC, total cholesterol; LDL-C, low density lipoprotein cholesterol; HDL-C, high density lipoprotein cholesterol; eGFR, estimated glomerular filtration rate; hs-CRP, high-sensitivity C-reactive protein; MA_ADP_, Maximum amplitude of ADP-induced clot strength; ADP_i_, ADP-induced platelet inhibition rate.

A total of 1,334 participants were split into four quartiles based on C1q: Q1 (0, 151.1], Q2 (151.1, 168.1], Q3 (168.1, 191.9], and Q4 (191.9, ~). **Table 2** shows covariates of interest and detects differences in patients with different C1q quartiles. Correlations between individual covariates of interest and correlations between C1q and TEG parameters were assessed using Pearson test or Spearman test as appropriate. **Figure 2** shows statistically significant correlation (p-value < 0.05) and correlation coefficient r. C1q has weak but significant positive associations with female patients, history of hypertension, WBC, PLT, TC, LDL-C, hs-CRP, and MA_ADP_, while correlating negatively with age and ADP_i_.

**Table 2:** Covariates of interest in patients with different C1q levels.

Variables	Overall	Q1 (334)	Q2 (333)	Q3 (333)	Q4 (334)	*p*
Female, *n* (%)	361 (27.1%)	51 (15.3%)	81 (24.3%)	104 (31.2%)	125 (37.4%)	<0.001
Age (years), mean ± SD	61.28 ± 9.53	62.87 (9.35)	62.18 (9.47)	60.31 (9.39)	59.75 (9.58)	<0.001
Smoking						0.154
Current smoker, *n* (%)	343 (25.7%)	93 (27.8%)	88 (26.4%)	83 (24.9%)	79 (23.7%)	
Ex-smoker, *n* (%)	92 (6.9%)	22 (6.6%)	25 (7.5%)	31 (9.3%)	14 (4.2%)	
Never-smokers, *n* (%)	899 (67.4%)	219 (65.6%)	220 (66.1%)	219 (65.8%)	241 (72.2%)	
Hypertension, *n* (%)	828 (62.1%)	189 (56.6%)	204 (61.3%)	218 (65.5%)	217 (65.0%)	0.066
Diabetes, *n* (%)	470 (35.2%)	109 (32.6%)	119 (35.7%)	124 (37.2%)	118 (35.3%)	0.657
WBC (×10^9^/L), mean ± SD	6.72 ± 1.89	6.26 ± 1.73	6.74 ± 1.93	6.76 ± 1.89	7.11 ± 1.90	<0.001
Hb (g/L), mean ± SD	140.52 ± 15.97	140.84 ± 15.97	139.77 ± 15.49	139.76 ± 15.82	141.72 ± 16.56	0.320
PLT (×10^9^/L), mean ± SD	212.71 ± 3.86	193.41 ± 50.83	209.76 ± 51.93	219.37 ± 50.82	228.33 ± 55.64	<0.001
TC (mmol/L), mean ± SD	4.01 ± 0.98	3.75 ± 0.85	3.92 ± 1.02	4.04 ± 0.87	4.33 ± 1.07	<0.001
LDL-C (mmol/L), mean ± SD	2.34 ± 0.80	2.15 ± 0.76	2.246 ± 0.78	2.36 ± 0.72	2.58 ± 0.89	<0.001
HDL-C (mmol/L), mean ± SD	1.09 ± 0.25	1.09 ± 0.24	1.07 ± 0.26	1.094 ± 0.26	1.08 ± 0.25	0.661
eGFR (ml/min/1.73 m^2^), median [IQR]	94.57 [85.90–101.23]	95.52 [86.91–101.75]	94.40 [86.54–100.69]	94.49 [84.19–101.70]	94.33 [85.82–101.00]	0.834
hs-CRP (mg/L), median [IQR]	1.18 [0.50–2.93]	0.61 [0.30–1.58]	1.11 [0.4450–2.6500]	1.39 [0.62–3.08]	1.78 [0.86–4.12]	<0.001
C1q (mg/L), mean ± SD	172.41 ± 31.75	136.03 ± 11.34	159.74 ± 5.10	179.23 ± 7.21	214.60 ± 22.57	<0.001
MA_ADP_ (mm), median [IQR]	43.45 [34.98–51.30]	39.55 [32.33–48.10]	42.80 [33.30–50.95]	45.00 [37.00–52.65]	46.60 [37.48–54.73]	0.012
ADP_i_ (%), mean ± SD	37.15 ± 23.60	39.34 ± 21.73	38.61 ± 24.54	36.02 ± 23.71	34.62 ± 24.12	0.033
HRPR, *n* (%)	516 (38.7%)	88 (26.3%)	128 (38.4%)	144 (43.2%)	156 (46.7%)	<0.001

HRPR, high residual platelet reactivity; PLT, platelet count; Hb, hemoglobin; WBC, white blood cell count; TC, total cholesterol; LDL-C, low-density lipoprotein cholesterol; HDL-C, high-density lipoprotein cholesterol; eGFR, estimated glomerular filtration rate; hs-CRP, high-sensitivity C-reactive protein; MA_ADP_, maximum amplitude of ADP-induced clot strength; ADP_i_, ADP-induced platelet inhibition rate.

### TEG parameters in patients with different C1q levels

**Figure 3:**
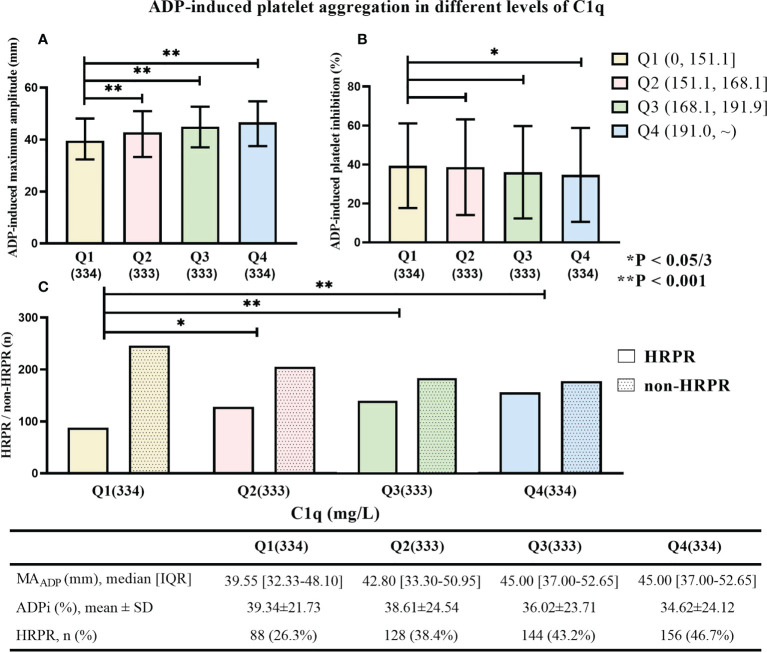
**(A)** ADP-induced platelet inhibition rate (ADP_i_), **(B)** the maximum amplitude of ADP-induced clot strength (MA_ADP_), and **(C)** the frequency of HRPR in patients with different C1q quartile. * means “P < 0.05/3”. ** means “P < 0.001”.

**Figure 3** displays monotonic variation of increased MA_ADP_ and decreased ADP_i_ with the increase of C1q level (median value of MA_ADP_ in each quartile: 39.60 mm, 42.85 mm, 45.10 mm, and 46.40 mm; mean value of ADP_i_ in each quartile: 39.28%, 38.74%, 35.71%, and 34.75%). Additionally, patients with higher C1q level were more likely to perform HRPR (Q1: 26.1%, Q2: 38.4%, Q3: 43.8%, and Q4: 46.4%). MA_ADP_ and frequency of HRPR in each higher C1q quartile significantly increase compared to the reference group (all p-values ≤ 0.001), but the significant difference in ADP_i_ is present only between the lowest and the highest quartile of C1q (p = 0.011).

### Effect of elevated C1q and other factors on antiplatelet therapy with clopidogrel

C1q quartile was set as an ordered categorical variable, which was included in a univariate logistic regression model. The results show that higher C1q level significantly increases risk of HRPR during clopidogrel therapy compared with the lowest quartile (Q2: OR = 1.745, 95% CI 1.257–2.425, p = 0.001; Q3: OR = 2.130, 95% CI 1.537–2.951, p < 0.001; Q4: OR = 2.450, 95% CI 1.771–3.390, p < 0.001). We constructed three additional multivariate logistic regression models (as described previously) to adjust for confounding variables. As **Table 3** shows, elevated C1q level remains to be an independent risk factor of HRPR during clopidogrel therapy (all p of each quartile in models 1, 2, and 3 < 0.05). When C1q was included as a continuous covariate in the logistic regression model, crude odds ratio and adjusted odds ratio were greater than 1. Furthermore, significant upward trends are observed regardless of univariate or multivariate logistic regression models (all p for trend < 0.05). In **Table 4**, we provided some of the ORs of all variables included in the multivariate analysis. After the multiple regression analysis, age, diabetes, and high PLT were independent factors associated with HRPR.

**Table 3:** Odds ratio (OR) and 95% CI for HRPR in each C1q quartile.

C1q, mg/L	Non-HRPR	HRPR	Crude OR (95% CI)	OR (95% CI) in model 1	OR (95% CI) in model 2	OR (95% CI)^1^ in model 3
C1q	-	-	1.010 (1.006–1.014)	1.009 (1.005–1.013)	1.009 (1.005–1.013)	1.009 (1.005–1.014)
Q1, ≤151.1	246	88	-	-		-
Q2, (151.1, 168.1]	205	128	1.745 (1.257–2.425)	1.678 (1.199–2.349)	1.668 (1.191–2.337)	1.722 (1.215–2.440)
Q3, (168.1, 191.9]	189	144	2.130 (1.537–2.951)	2.047 (1.459–2.872)	2.030 (1.446–2.851)	2.015 (1.413–2.874)
Q4, >191.9	178	156	2.450 (1.771–3.390)	2.286 (1.626–3.214)	2.279 (1.619–3.207)	2.362 (1.631–3.421)
*p* for trend^2^			<0.001	<0.001	<0.001	<0.001

^1^Odds ratio (OR) and 95% CI for HRPR in each C1q quartile were calculated using univariate and multivariable logistic regression. Model 1 was adjusted for age, sex (female), smoking status (current smoker), and medical history of diabetes and hypertension. Model 2 was adjusted for variables included in Model 1, smoking status (current smoker), and medical history of diabetes and hypertension. Model 3 was adjusted for variables included in Model 2 and laboratory data including PLT, WBC, Hb, LDL-C, HDL-C, eGFR, and hs-CRP. ^2^Test for trend based on variable containing median value for each quintile. HRPR, high residual platelet reactivity; OR, odds ratio; CI, confidence interval; PLT, platelet count; Hb, hemoglobin; WBC, white blood cell count; LDL-C, low-density lipoprotein cholesterol; HDL-C, high-density lipoprotein cholesterol; eGFR, estimated glomerular filtration rate; hs-CRP, high-sensitivity C-reactive protein.

**Table 4:** ORs (and 95% CIs) for HRPR of other variables.

variables	OR (95% CI) in model 1	OR (95% CI) in model 2	OR (95% CI) in model 3
Age	1.026 (1.013–1.039)	1.026 (1.013–1.040)	1.023 (1.008–1.040)
Sex	2.212 (1.708–2.863)	2.236 (1.706–2.932)	1.372 (0.991–1.898)
Current smoker	-	1.117 (0.840–1.486)	1.184 (0.880–1.592)
Diabetes	-	1.235 (0.971–1.571)	1.290 (1.004–1.658)
Hypertension	-	1.056 (0.830–1.345)	1.092 (0.849–1.405)
PLT	-	-	1.006 (1.004–1.009)
WBC	-	-	0.807 (0.749–0.870)
Hb	-	-	0.984 (0.975–0.993)
LDL-C	-	-	1.101 (0.943–1.285)
HDL-C	-	-	1.084 (0.654–1.797)
eGFR	-	-	0.999 (0.990–1.008)
hsCRP	-	-	1.023 (0.996–1.052)

ORs (and 95% CIs) for HRPR of other variables were calculated using univariate and multivariable logistic regression. HRPR, high residual platelet reactivity; OR, odds ratio; CI, confidence interval; PLT, platelet count; Hb, hemoglobin; WBC, white blood cell count; LDL-C, low-density lipoprotein cholesterol; HDL-C, high-density lipoprotein cholesterol; eGFR, estimated glomerular filtration rate; hs-CRP, high-sensitivity C-reactive protein.

### Restrictive cubic spline

**Figure 4:**
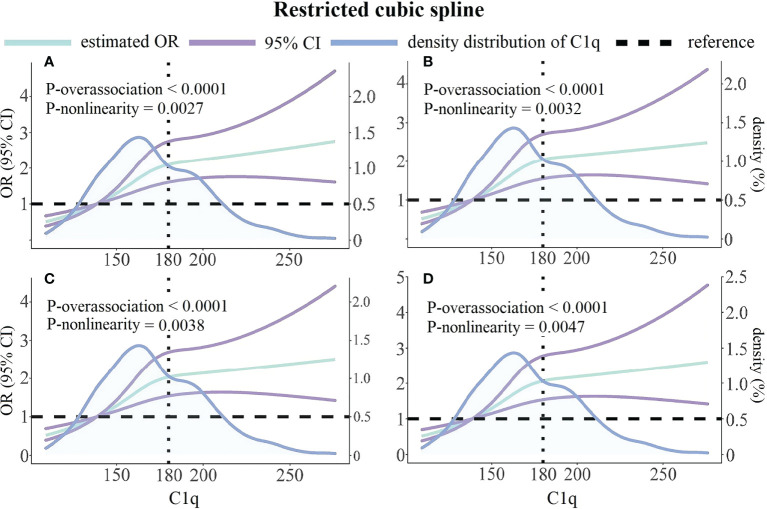
Unadjusted and multivariable-adjusted ORs and 95% CIs for HRPR according to C1q and the density distribution curve of C1q. **(A)** Unadjusted model. **(B)** Adjusted for variables included in Model 1 as **Table 3** described. **(C)** Adjusted for variables included in Model 2 as **Table 3** described. **(D)** Adjusted for variables included in Model 3 as **Table 3** described. Green solid lines represent ORs, with purple lines representing the 95% CIs. Black horizontal dotted lines represent the references corresponding to an OR of 1.0. Blue solid lines describe density distribution of C1q. Reference point is the median of C1q’s first quartile with three knots placed at the 25th, 50th and 75th percentiles of C1q. OR, odds; CI, confidence interval.

We constructed restrictive cubic spline models to flexibly visualize the relationship between the risk of HRPR during clopidogrel therapy and serum C1q on a continuous scale with or without correcting for covariates. In **Figure 4**, inverted L-shaped RCS curve indicates a non-linear relationship (p for non-linear < 0.05). The crude and adjusted odds ratios for HRPR (the median of C1q’s first quartile was set as reference value: 138.7 mg/L) rise with the increase of C1q until approximately 180 mg/L of C1q, then the curves are relatively gentle and the slope changes little.

## Discussion

We reported the following novel findings based on the present cross-sectional study. Serum level of C1q is correlated with ADP-induced platelet aggregation assessed by TEG parameters in CAD patients receiving clopidogrel therapy and elevated C1q was proved to be an independent risk factor for HRPR induced by ADP during clopidogrel treatment. Besides these, there exist weak but significant associations between C1q and several HRPR-related factors detected in current and previous studies. Previous studies had demonstrated that the occurrence of HRPR significantly increases the risk of thrombotic and ischemic events in CAD patients (26, 27), which might suggest the potential predictive value of C1q in predicting certain adverse cardiovascular events. Based on the above findings, C1q emerges as a convenient, accessible, and reliable predictor for HRPR induced by ADP during clopidogrel treatment.

Antiplatelet therapy represents the cornerstone of secondary prevention for avoiding coronary artery thrombosis in patients with CAD. Although novel potent P2Y12 receptor antagonists including prasugrel and ticagrelor had already been widely applied in clinical practice, clopidogrel is still indispensable in many clinical scenarios due to its advantages in lower bleeding risk, lower economic burden, and better medication adherence (2, 28). As a prodrug, clopidogrel exerts its effect after being metabolized by cytochrome P450 (CYP450). There is a higher incidence of HRPR in east Asians, due to the higher prevalence of the CYP2C19 loss-of-function alleles (29), and the incidence in the current study population is 38.7%. The genetic factor is a crucial, but not a unique factor affecting antiplatelet treatment with clopidogrel (30), and the routine detection of HRPR-related genotypes increases healthcare costs. Therefore, it is essential to investigate other potential risk factors of HRPR. Contemporary biomedical literature supports that inflammation may play an important role in the progression of atherosclerosis and thrombosis initiated by rupture of atherosclerotic plaques, and a previous study found that inflammatory biomarkers could influence ADP-induced platelet aggregation assessed by platelet function test during clopidogrel treatment (31). As a major participant in innate immune responses and inflammatory processes, the complement system is also involved in the regulation of atherosclerosis. C1q can induce cytokines and enhance NLRP3 inflammasome to promote atherosclerosis development by initiating a classical pathway (32) while retarding the formation of the necrotic core in plaques via promoting cholesterol efflux from macrophage foam cells and downregulating their apoptosis (33). Despite evidence that C1q and downstream complement components in the classical pathway interact with platelets to activate platelets and induce thrombosis (18), no former studies had explored the effect of C1q on antiplatelet therapy with clopidogrel.

TEG is a dynamic observation of the blood coagulation process by dynamic measurement of clot strength. MA_ADP_ represents the peak clot strength in citrated or heparinized whole blood samples with the addition of moderate ADP (23), which is used to evaluate the aggregation capacity of both platelet and fibrin induced by ADP. The current consensus sets MA_ADP_ > 47 mm as a predictive factor of ischemic events in post-PCI patients with DAPT (34). The efficacy of clopidogrel in reducing ADP-induced platelet aggregation is assessed using ADP_i_, which is calculated according to the formula: ${\text{ADP}}_{\text{i}}=\frac{\left({\text{MA}}_{\text{ADP}}–{\text{MA}}_{\text{Fibrin}}\right)}{\left({\text{MA}}_{\text{Thrombin}}–{\text{MA}}_{\text{Fibrin}}\right)}\times 100\%$ . We established a composite criterion that defined patients with a MA_ADP_ of >47 mm plus an ADP_i_ of <50% as patients with HRPR based on previous literature (24, 25). Our findings show that serum C1q is significantly positively related to MA_ADP_ as well as negatively related to ADP_i_. Compared to MA_ADP_, ADP_i_ shows a much weaker association with C1q, which is demonstrated by a smaller correlation coefficient and only one significant difference of ADP_i_ between the lowest and the highest quartile of C1q. At the same time, HRPR risk exhibits an inverted L-shaped curve pattern with an increase in serum C1q.

The explanations for current findings are unclear but may be multifactorial. Consistent with previous literature (30, 35), current research identified several HRPR-related factors, including aging, sex, worse renal function, and diabetes. The correlations of C1q with some of these factors might partially explain our findings. Despite adjusting for potential mediator variables and confounders, elevated C1q is still an independent indicator of platelet aggregation assessed by TEG parameters, which implies that complement components themselves affected ADP-induced platelet activity in CAD patients treated by clopidogrel. As a prodrug, clopidogrel needs to be metabolized by CYP450 to execute its antiplatelet function. Elevated inflammatory cytokines such as interleukin-6 (IL-6) and the tumor necrosis factor-alpha (TNF-α) downregulate the expression and activity of CYP450 in the state of inflammation (36, 37). We propose a hypothetical explanation that C1q may cause pharmacokinetic effects on CYP2C19-mediated clopidogrel metabolism by participating in the inflammatory process. Previous evidence that C1q enhances the release of inflammatory cytokines including TNF-α and IL-6 might partially support our hypothesis (38, 39). P-glycoprotein, a transmembrane transporter encoded by the ABCB1 gene, can expel drugs (including clopidogrel) from the cell interior. ABCB1 polymorphisms and abnormal activity of P-glycoprotein affect clopidogrel’s absorption and transport, and TRITON-TIMI 38 trail (40) had detected that the ABCB1 3435 TT genotype attenuates platelet inhibition and increases the risk of recurrent ischemic events in acute coronary syndrome patients receiving clopidogrel treatment. In the field of oncology, some literature had found that WNT/β-catenin canonical signaling pathway positively regulates the ABCB1 gene expression in tumor cells (41). Naito et al. (42) provided evidence demonstrating complement C1q as an activator of Wnt signaling. These existing findings suggest a plausible mechanism that elevated C1q affects clopidogrel absorption and transport via activating Wnt signaling to promote p-glycoprotein expression. Besides these, C1q binding to C1q receptors (gC1qR/p33 and cC1qR) expressed on the platelet surface can enhance platelet aggregation and activation (18). After ADP activating platelets via binding to P2Y12 and P2Y1 receptors, platelet degranulation and the release of P-selectin from alpha granules are upregulated. P-selectin localized on the platelet membrane binds P-selectin glycoprotein ligand-1 (PSGL-1) on leukocytes to recruit leukocytes, which enhances the pro-inflammatory and pro-thrombotic effects of leukocytes (43). In vitro experiments had found that clopidogrel downregulates ADP-induced P-selectin expression and platelet–leukocyte adhesion (44). C1q binding to C1q receptors can promote the release of P-selectin, then initiate downstream events (18), which is another possible mechanism. Stable thrombosis is formed by platelets and a cross-linked fibrin network. Coagulation is a complex cascade involving activation of the coagulation system, activation of platelet, and formation of cross-linked fibrin, all steps of which interact with each other. The effects of C1q on thrombosis are not restricted to platelet activation and aggregation and involve cross-linking of fibrin. As to why MA_ADP_, a TEG parameter evaluating aggregation capacity of both platelet and fibrin, has a stronger association with C1q than ADP_i_, one explanation may be that C1q directly or indirectly affects cross-linking of fibrin. Considering the above, despite these findings we detected, the exact mechanism remains speculation based on previous research.

In this study, we had tried to avoid effects on C1q level of identifiable inflammation by just recruiting stable CAD patients and setting a series of exclusion criteria. Among the study population, elevated C1q significantly promotes ADP-induced thrombosis measured by TEG parameters and independently predicted the risk of HRPR during clopidogrel therapy. Our findings call for more attention to C1q when physicians formulate antiplatelet prescriptions and evaluate future thrombosis risk for SCAD patients undergoing PCI.

Several limitations of the current study should be acknowledged. Our study was an observational analysis derived from a small single-center sample, which might cause potential bias and limit the extrapolation of our conclusions. Second, we were unable to record some related factors, such as patients’ HRPR-related genotypes, due to limited resources. Third, our cross-sectional study had not analyzed post-discharge follow-up adverse cardiovascular events, which limit clinical application of current findings. We plan to investigate whether C1q could increase the risk of thrombotic events via affecting ADP-induced platelet activity in our future, follow-up study, when we complete a protocol for a detailed, comprehensive study. Fourth, TEG is based on an in vitro coagulation process and not the gold standard for HRPR, which might not make an entirely accurate evaluation of the actual in vivo platelet aggregation. Fifth, both TEG parameters and other laboratory information were measured at baseline only once. Sixth, due to technical limitations in our Department of Laboratory Medicine, we evaluated platelet function by only measuring platelet aggregation. We plan to use more methods to evaluated platelets’ other functions during clopidogrel therapy in future basic research. Finally, the biological mechanism underlying our conclusion remains unclear, and the detailed mechanism needs further investigation.

## Conclusion

The current study firstly indicated that elevated C1q promotes TEG-simulated platelet aggregation induced by ADP in CAD patients during clopidogrel therapy and elevated C1q is an independent risk factor of clopidogrel HRPR. Our finding implied that elevated C1q is a novel, accessible, and reliable clinical biomarker used for risk assessment of clopidogrel HRPR and to help guide the use of antiplatelet treatment.

## Data availability statement

The raw data supporting the conclusions of this article will be made available by the authors, without undue reservation.

## Ethics statement

The studies involving human participants were reviewed and approved by Beijing Anzhen Hospital, Capital Medical University. Written informed consent for participation was not required for this study in accordance with the national legislation and the institutional requirements.

## Author contributions

ZZ designed the study, analyzed the data, and drafted the manuscript. MM and XH analyzed the data and drew the figures. KH and TS collected the data from the electronic medical system and constructed the database. SY and YZ designed the study and revised the manuscript. All authors contributed to the article and approved the submitted version.

## Funding

This work was supported by the grant from National Key Research and Development Program of China (2017YFC0908800), Beijing Municipal Administration of Hospitals’ Mission plan (SML20180601), Capital’s Funds for Health Improvement and Research (CFH 2020-2-2063), and Beijing Municipal Natural Science Foundation (7202041).

## Conflict of interest

The authors declare that the research was conducted in the absence of any commercial or financial relationships that could be construed as a potential conflict of interest.

## Publisher’s note

All claims expressed in this article are solely those of the authors and do not necessarily represent those of their affiliated organizations, or those of the publisher, the editors and the reviewers. Any product that may be evaluated in this article, or claim that may be made by its manufacturer, is not guaranteed or endorsed by the publisher.
