# Supplementary material for: Elevated serum C1q is an independent predictor of high residual platelet reactivity in CAD patients receiving clopidogrel therapy
Source: Front Immunol. 2022 Sep 30;13:969984. doi: 10.3389/fimmu.2022.969984 (PMC9561631; doi:10.3389/fimmu.2022.969984)
Supplement: Supplementary file 1 [file Table_1.docx]

Supplementary Material

# Supplementary Table

VIF for each covariate in multivariate logistic regression

| covariate | VIF | | |
| --- | --- | --- | --- |
|  | Model 1 | Model 2 | Model 3 |
| C1q | 1.063 | 1.067 | 1.218 |
| age | 1.067 | 1.112 | 1.558 |
| sex | 1.087 | 1.180 | 1.610 |
| current smoker | - | 1.147 | 1.180 |
| diabetes | - | 1.023 | 1.056 |
| hypertension | - | 1.032 | 1.069 |
| PLT | - | - | 1.226 |
| WBC | - | - | 1.181 |
| Hb | - | - | 1.485 |
| LDL-C | - | - | 1.132 |
| HDL-C | - | - | 1.166 |
| eGFR | - | - | 1.488 |
| hs-CRP | - | - | 1.135 |

VIF, variance inflation factor; PLT, platelet count; Hb, hemoglobin; WBC, white blood cell count; LDL-C, low density lipoprotein cholesterol; HDL-C, high density lipoprotein cholesterol; eGFR, estimated glomerular filtration rate; hs-CRP, high-sensitivity C-reactive protein rate
